# Comparisons among rainbow trout, *Oncorhynchus mykiss,* populations of maternal transcript profile associated with egg viability

**DOI:** 10.1186/s12864-021-07773-1

**Published:** 2021-06-15

**Authors:** Gregory M. Weber, Jill Birkett, Kyle Martin, Doug Dixon, Guangtu Gao, Timothy D. Leeds, Roger L. Vallejo, Hao Ma

**Affiliations:** 1 USDA/ARS National Center for Cool and Cold Water Aquaculture, Kearneysville, WV USA; 2 Troutlodge Inc., Sumner, WA USA; 3 USDA/ARS Ruminant Diseases and Immunology Research Unit, National Animal Disease Center, Ames, IA USA

**Keywords:** Rainbow trout, Egg quality, mRNA, Maternal RNA, Mitochondria

## Abstract

**Background:**

Transcription is arrested in the late stage oocyte and therefore the maternal transcriptome stored in the oocyte provides nearly all the mRNA required for oocyte maturation, fertilization, and early cleavage of the embryo. The transcriptome of the unfertilized egg, therefore, has potential to provide markers for predictors of egg quality and diagnosing problems with embryo production encountered by fish hatcheries. Although levels of specific transcripts have been shown to associate with measures of egg quality, these differentially expressed genes (DEGs) have not been consistent among studies. The present study compares differences in select transcripts among unfertilized rainbow trout eggs of different quality based on eyeing rate, among 2 year classes of the same line (A1, A2) and a population from a different hatchery (B). The study compared 65 transcripts previously reported to be differentially expressed with egg quality in rainbow trout.

**Results:**

There were 32 transcripts identified as DEGs among the three groups by regression analysis. Group A1 had the most DEGs, 26; A2 had 15, 14 of which were shared with A1; and B had 12, 7 of which overlapped with A1 or A2. Six transcripts were found in all three groups, *dcaf11*, *impa2*, *mrpl39_like*, *senp7*, *tfip11* and *uchl1*.

**Conclusions:**

Our results confirmed maternal transcripts found to be differentially expressed between low- and high-quality eggs in one population of rainbow trout can often be found to overlap with DEGs in other populations. The transcripts differentially expressed with egg quality remain consistent among year classes of the same line. Greater similarity in dysregulated transcripts within year classes of the same line than among lines suggests patterns of transcriptome dysregulation may provide insight into causes of decreased viability within a hatchery population. Although many DEGs were identified, for each of the genes there is considerable variability in transcript abundance among eggs of similar quality and low correlations between transcript abundance and eyeing rate, making it highly improbable to predict the quality of a single batch of eggs based on transcript abundance of just a few genes.

**Supplementary Information:**

The online version contains supplementary material available at 10.1186/s12864-021-07773-1.

## Background

Egg quality is fundamental to reliable seed stock production in aquaculture and yet what makes an egg developmentally competent to be fertilized and subsequently develop into a normal embryo is poorly understood [1–3]. Fertilization rates are often high in the rainbow trout industry but the quality of eggs in fishes can be affected by intrinsic factors such as the genetics and age of the brood fish [1, 4–10] and extrinsic factors that can vary with hatchery environments and practices [11–15]. Female rainbow trout broodstock do not volitionally oviposit in captivity and therefore must be stripped of their eggs following ovulation. The female gamete obtained by this stripping process or when spawning naturally is an oocyte arrested in metaphase of the second meiotic division that should be competent for fertilization. The oocyte is largely transcriptionally silent from the end of oocyte growth until the zygote genome is activated, referred to as zygotic genome activation (ZGA), which begins at about the mid-blastula transition (MBT) in most vertebrates. The oocyte therefore serves as a reservoir for RNAs as well as other biomolecules including proteins and lipids accumulated during oogenesis, for utilization from oocyte maturation through early embryonic development [16, 17]. Levels of biomolecules in the egg including proteins, lipids, and RNAs have been linked to egg viability in many fishes including rainbow trout [1–3, 18].

The almost total reliance of the late stage oocyte and early embryo on maternally derived RNAs has led to investigations of associations between the maternal transcriptome and measures of developmental competence in several species of fish and has been reviewed [3, 19, 20]. Most investigations identified mRNAs that reflect differences in egg quality by simply comparing transcript expression profiles among eggs or early embryos exhibiting variation in measures of developmental competence, usually including progression to a specific developmental stage or a developmental abnormality [21–27]. A number of studies, primarily in rainbow trout, have identified mRNAs differentially expressed among eggs of different quality in response to treatments used to alter time of spawning through photoperiod manipulation or hormone treatment [15, 28] and in response to being overripe due to post-ovulatory aging [29, 30]. In addition to mRNAs, profiles of microRNAs and mitochondrial genome-encoded small RNAs were related to egg deterioration caused by post-ovulatory aging in rainbow trout [31, 32]. Recently, we identified over 1000 differentially expressed transcripts or genes (DEGs) in unfertilized rainbow trout eggs that are associated with eyeing rate [27]. However, these differences were only found when the libraries used for sequencing were prepared following polyadenylation capture and not rRNA-removal, suggesting differences in egg quality may derive in part from differences in maternal transcript activation and cytoplasmic polyadenylation before ovulation.

Much has been learned about the contribution of maternal mRNAs to egg quality in fish. As expected, many of the transcripts that appear dysregulated in poor quality eggs are in pathways known to be involved in critical processes taking place at the developmental stages investigated [3, 19, 20]. Nevertheless, there is considerable disparity in DEGs identified among the studies. This may be due to differences in species, stages investigated, measures of egg quality, intrinsic and extrinsic causes of the differences in quality, and molecular and statistical approaches employed. Furthermore, studies thus far have focused on identifying possible DEGs for dysregulation but compared transcriptomes of few individuals. The aim of the present study is to further evaluate the robustness of genes identified as possible markers of egg quality using a commercially important species, rainbow trout. To meet this aim we designed an assay based on the nCounter analysis data system (Nanostrings Technologies; Seattle, WA) to compare expression of 65 mRNAs previously identified as being differentially expressed with egg quality (Additional file 1: Table S1). The nCounter analysis data system was chosen in part because it is relatively easy to customize the multiplex CodeSet to update or meet specific needs of the user. Most of the genes incorporated in the assay are DEGs from our previous transcriptome analysis of egg viability in rainbow trout using RNA-Seq, [27], but also includes 10 additional transcripts reported as dysregulated in poor quality eggs in rainbow trout [28–30], and also *igf-3* since many IGF-system genes were already in the assay. The genes from [27] were selected for the assay primarily based on magnitude of statistical differences and fold-change. Three populations of broodstock were compared including two different year classes from a commercial line, referred to as Group A1 and A2 respectively, and females from the 2015 year-class at the National Center for Cool and Cold Water Aquaculture (NCCCWA) referred to as Group B. One of the year classes from the commercial line, Group A1, included eggs from the same females used in our RNA-Seq study [27]. In all 152 families were included in the study. The present study had four aims. The first aim (i) was to determine if DEGs identified in a limited number of fish were DEGs in a broader sample; the second aim (ii) was to determine if the identified DEGs were consistent year to year within the same line; the third aim (iii) was to determine if they varied from line to line, and the fourth aim (iv) was to determine if a small set of genes can act as a reliable universal marker for egg quality in rainbow trout.

## Results

### Eyeing rate and early embryo viability

**Fig. 1:**
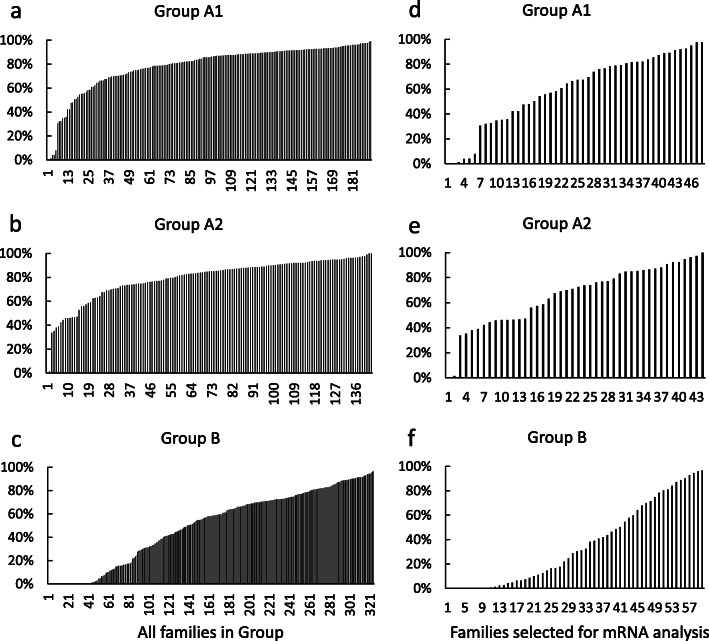
Eyeing rates of all the surveyed rainbow trout families in the breeding groups (**a**-**c**) and those selected for mRNA analysis (**d**-**f**)

Eyeing rate was assessed at ~ 250 accumulated temperature units (ATUs) post fertilization. This timepoint is slightly after retinal pigmentation but often used by hatcheries because embryos are resistant to handling or mechanical shock [33, 34], most of embryonic mortality has already occurred [35], and it still allows time for dead and subviable egg removal and shipment to hatching facilities. Eyeing rates were collected for all families in each of the broodstocks that made up that year’s cohort for genetic selection for that line. A total of 192, 143, and 325 families were evaluated for Groups A1, A2, and B respectively, with mean eyeing rates of 78.3% + 0.015, 79.1% + 0.015, and 49.7 + 0.017 (Fig. 1abc). The data on eyeing rate for Group A1 were previously presented in Ma et al. [27]. Historical eyeing rates are higher for the commercial hatchery lines from which groups A1 and A2 were collected, than for the NCCCWA line from which group B was collected. Nevertheless, there were fewer egg lots with survival less than 30% than has usually been observed (Kyle Martin, personal communication) with only 6 and 2 families yielding eyeing rates below 30% in Groups A1 and A2 respectively, and all these were below 10%. Transcript abundance analysis was determined for 48, 44, and 60 families for Groups A1, A2 and B respectively including all families with less than 30% eyeing in Groups A1 and A2 (Fig. 1def).

Families from Group A1 with eyeing rates under 80% were generated with sperm that also generated families with eyeing rates over 78%, supporting subfertility was due to the eggs and not the sperm. Sperm used in Group A2 to fertilize each of the 27 families with eyeing rates between 20 and 80% also produced families with eggs from a different female that yielded 22 families with eyeing rates over 70% and 18 over 80% support eyeing rates were mainly due to egg quality. Although the sperm lot used to produce the family with an eyeing rate of 0% used in the present study also yielded a family with an eyeing rate of 83.1%, sperm from the family with 1.4% eyeing in the present study was not used to produce a second family making it unclear if the low eyeing rate was due to the quality of the egg or sperm, although normalized read values are consistent with reduced egg quality (Additional file 1: Table S5B). Sperm quality could not be ruled out as contributing to eyeing rates in Group B since sires were only used once. Egg lots used in the study showed no obvious visible signs of poor quality including overripening when examined before fertilization.

Mortality before eyeing has been previously investigated in line A including for 20 of the families used in Group A1, and found to predominantly take place before the 32-cell stage [27, 36]. In the present study embryo cleavage was assessed at about 19–20 h post fertilization at ~ 10 °C and early embryo development or streak rate was estimated at about 10 days post fertilization for the 60 families in Group B (Table 1; Additional file 1: Table S2). Fertilization rate was high with families averaging 89.6% of zygotes completing first cleavage. The majority of the zygotes of families with eyeing rates greater than 80%, which we consider families with high quality eggs, reached at least the 16-cell stage, 91.6%, with some reaching the 32-cell stage, 43.2%. Those zygotes not reaching the 8-cell stage were therefore considered subviable and on average 76.7% of zygotes reached this stage. This is well above the mean eyeing rate of 35.9%. We prefer assessing early stage mortality after most of the embryos in the families with greater than 80% eyeing rates reach the 32-cell stage, which we failed to meet in Group B samples. We therefore included a measure of streak rate which as evaluated is only a rough estimate of development to an elongating embryo. The average streak rate among the families was 63.7% which is still well above the eyeing rate supporting mortality was taking place throughout development to eyeing in Group B.

**Table 1:** Assessment of early embryo development in Group B selected families

		Embryos collected at ~ 20 h post fertilization						
	Families	Embryos with > 2 cells (%)	Embryos with > 4 cells (%)	Embryos with > 8 cells (%)	Embryos with > 16 cells (%)	Embryos with > 32 cells (%)	Streak rate (%)	Eyeing rate (%)
All families	60	89.6	78.5	76.7	65.7	17.6	63.7	35.9
Eyeing rate > 80%	10	98.0	96.8	96.8	91.6	43.2	97.1	89.2

The percentage of embryos reaching each cell stage by ~ 20 h post fertilization, and streak and eyeing rate, are indicated

### Transcriptome abundance analysis

**Fig. 2:**
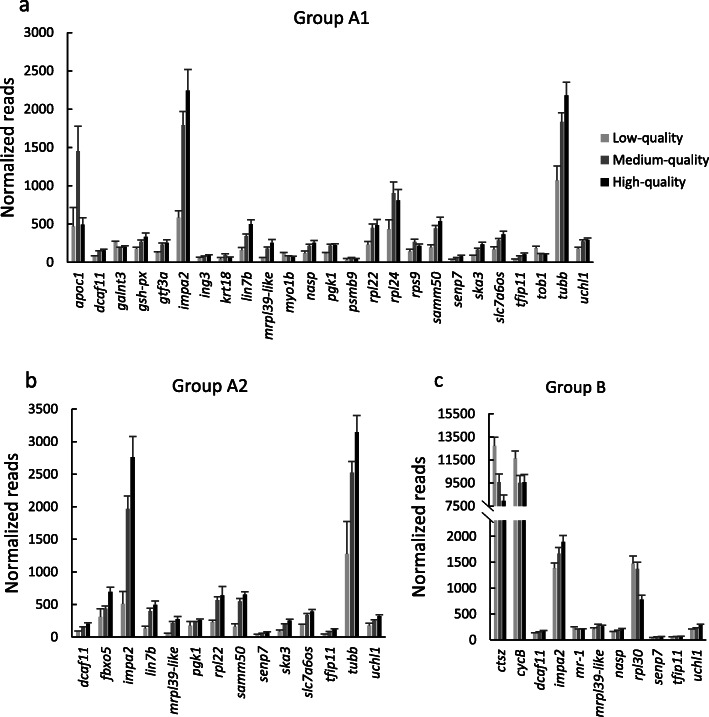
Normalized reads for nuclear genes that were differentially expressed with egg quality. **a**) Group A1. **b**) Group A2. **c**) Group B. Values are mean + SEM for families designated as having low-quality, medium-quality, or high-quality eggs based on eyeing rates

Overall, there were 32 transcripts identified as DEGs among the three populations or groups by regression analysis (Tables 2, 3, 4; Fig. 2a-c; Additional file 1: Tables S3AB). More DEGs were shared between Groups A1 and A2 which were within the same line, than between these groups and Group B which is from a different line. Group A1 (Table 2) had the most, 26; A2 (Table 3) had 15, 14 of which were shared with A1; and B (Table 4) had 12, 7 of which overlapped with A1 or A2. Six transcripts, all from nuclear genes, were found to be differentially expressed in all groups (Fig. 2a-c; Table 5). Low raw read counts limited the detection of differences in the same 10 genes in each of the three groups and two additional genes among the groups (Additional file 1: Tables S4A-D).

**Table 2:** Group A1 Normalized reads

	Low quality		Medium quality		High quality					
Gene	Mean	SEM	Mean	SEM	Mean	SEM	Mean reads	RSQ	RC	*P* value
Mitochondrial genes										
*mt-atp8*	64,319	5561	90,104	10,130	108,669	14,165	92,682	0.0848	515.0	0.0717
*mt-co1*	162,344	27,574	258,866	31,556	295,392	46,645	258,215	0.0255	899.0	0.2100
*mt-cytb*	91,006	18,224	149,496	16,174	167,933	24,250	147,946	0.0739	799.3	**0.0426**
*mt-nd4l*	6761	570	8704	954	9877	1308	8828	0.0663	41.9	0.1839
*mt-dlp*	12,706	1006	20,170	2665	25,906	4547	21,029	0.0178	67.5	0.2258
Nuclear genes										
*agfg1-like*	57.4	2.9	61.6	6.7	66.8	8.2	62.7	0.0036	0.0652	0.7895
*anxa2*	48.2	5.1	67.8	7.0	78.0	9.5	68.5	0.0476	0.2650	0.1173
*apoc1*	468.5	247.2	1450.8	329.0	492.0	90.9	1028.4	0.0152	5.8891	**0.0433**
*atg16l1*	39.3	6.4	62.6	6.9	70.0	10.2	62.0	0.0408	0.2489	0.0947
*bmp10-like*	27.1	4.5	38.1	4.0	45.0	5.5	38.9	0.0547	0.1650	ND
*ctsz*	15,381.5	2115.8	10,910.5	935.6	13,587.7	1600.5	12,306.0	0.0067	−15.5459	0.3711
*cycB*	11,733.3	2287.4	9881.1	444.9	11,933.6	795.4	10,754.0	0.0001	−1.1530	0.9792
*dcaf11*	80.6	8.1	140.1	11.9	153.4	16.1	136.8	0.1081	0.7006	**0.0068**
*dglucy*	11.7	2.3	13.5	0.6	11.9	1.0	12.8	0.0040	−0.0081	ND
*erich3*	14.0	2.0	13.5	0.6	12.0	1.0	13.1	0.0491	−0.0274	ND
*fbxo5*	325.8	55.0	446.7	44.0	563.5	70.0	468.0	0.0512	1.8846	0.0996
*galnt3*	261.8	14.6	188.2	11.5	201.6	15.7	201.6	0.0671	−0.5478	**0.0321**
*gsh-px*	175.4	21.8	266.6	24.7	333.2	49.4	276.0	0.0684	1.3579	**0.0205**
*gtf3a*	122.9	15.0	230.8	22.1	255.6	38.2	225.1	0.0961	1.3339	**0.0041**
*haus3*	144.1	28.8	210.9	20.3	237.3	31.3	210.8	0.0371	0.7217	0.1045
*hbb*	2926.0	1803.7	4606.3	845.1	1833.6	647.8	3529.8	0.0003	−2.4889	0.4535
*ifngr1*	15.2	2.8	13.5	0.6	12.3	1.0	13.3	0.0708	−0.0359	ND
*igf-1*	19.7	2.9	24.0	2.3	24.7	3.7	23.7	0.0025	0.0211	ND
*igf-2*	46.2	7.8	37.1	3.8	54.2	10.7	43.6	0.0025	0.0493	ND
*igf-3*	15.5	3.1	24.9	2.8	31.3	4.8	25.7	0.0587	0.1321	ND
*igfr1b*	65.5	7.9	86.8	8.3	89.4	9.7	84.9	0.0209	0.1958	0.2058
*il17rd*	328.9	14.3	285.6	24.2	303.5	26.6	296.6	0.0062	−0.3000	0.3318
*impa2*	583.6	90.5	1790.5	180.4	2242.2	274.9	1780.8	0.2017	16.0697	**<.0001**
*ing3*	56.7	8.8	75.7	6.5	90.0	6.9	77.8	0.0977	0.3409	**0.0099**
*itga7*	18.2	3.7	26.6	2.9	28.4	4.5	26.1	0.0125	0.0587	ND
*kmt5b*	59.2	3.4	61.0	6.0	59.3	4.9	60.2	0.0014	0.0333	0.9126
*krt18*	41.9	22.6	95.5	14.7	58.2	11.6	77.1	0.0453	0.4992	**0.0098**
*krt8*	53.6	25.4	73.0	14.0	42.7	6.4	61.1	0.0099	0.2094	0.2152
*lin7b*	160.8	32.6	343.1	29.4	499.4	58.2	369.1	0.2269	3.2817	**<.0001**
*mettl3*	11.7	2.3	13.5	0.6	11.9	1.0	12.8	0.0040	−0.0081	ND
*mr-1*	281.7	41.9	248.7	10.6	275.6	16.0	261.3	0.0001	−0.0212	0.9661
*mrpl39-like*	54.8	9.4	179.4	20.6	255.5	40.2	187.6	0.1605	1.8167	**0.0006**
*myo1b*	115.4	12.2	78.8	6.5	73.8	6.4	81.8	0.1093	−0.3735	**0.0108**
*nasp*	122.2	26.3	214.4	22.2	253.2	32.3	215.0	0.1182	1.4015	**0.0014**
*npm2*	12.1	2.2	13.5	0.6	11.9	1.0	12.8	0.0085	−0.0117	ND
*ntan1*	326.0	31.4	309.2	22.0	368.3	29.6	329.7	0.0030	0.2094	0.9207
*pde4d*	58.3	8.0	79.9	9.5	89.1	12.5	80.1	0.0132	0.1851	0.3982
*pgk1*	118.6	10.5	219.7	17.8	223.8	19.5	208.3	0.0992	0.9516	**0.0023**
*phb2*	60.2	12.0	63.6	6.0	69.0	7.3	64.9	0.0109	0.1067	0.4136
*psmb9*	32.5	19.5	53.4	8.3	40.7	7.5	46.8	0.0392	0.2712	**0.0029**
*ptgs2*	505.0	113.3	424.6	28.0	518.9	44.5	464.1	0.0181	0.8142	0.4600
*pyc*	64.4	4.8	68.2	6.9	71.9	8.0	68.9	0.0018	0.0461	0.8653
*ran*	22.5	3.6	34.4	2.9	33.5	4.3	32.6	0.0552	0.1220	ND
*rpl22*	230.7	39.3	448.7	52.3	484.2	74.6	432.5	0.0636	2.3447	**0.0040**
*rpl24*	434.3	122.4	903.7	146.1	810.8	139.7	816.0	0.0132	2.6224	**0.0473**
*rpl30*	563.4	180.3	1094.1	164.4	807.7	137.7	938.2	0.0136	2.9736	0.0538
*rplp1*	35.0	4.2	45.4	4.7	56.3	6.8	47.5	0.0400	0.1685	0.1600
*rps9*	142.3	27.2	264.5	37.4	214.9	24.7	233.7	0.0425	1.1405	**0.0227**
*s100a1*	115.1	14.3	160.7	18.0	177.8	27.8	160.3	0.0218	0.4758	0.3086
*samm50*	196.1	34.4	443.8	35.9	540.1	47.4	443.0	0.2166	3.2374	**<.0001**
*sec14l2*	48.4	5.9	74.1	8.1	87.4	10.4	75.0	0.0572	0.3331	0.0628
*senp7*	35.5	5.8	57.4	5.9	77.6	13.9	61.0	0.1255	0.4848	**0.0050**
*ska3*	84.0	8.6	167.6	15.3	233.7	28.4	177.8	0.1846	1.4286	**0.0005**
*slc7a6os*	169.4	35.4	285.5	25.8	364.0	42.2	295.5	0.1429	1.9438	**0.0026**
*smc6*	110.0	8.7	86.6	6.6	91.7	7.3	91.1	0.0313	−0.1914	0.1576
*tfip11*	38.8	4.4	79.1	7.2	103.8	16.0	81.8	0.1226	0.5800	**0.0007**
*tob1*	184.5	26.8	107.1	8.1	99.0	10.7	114.2	0.2126	−0.8219	**0.0004**
*tubb*	1071.7	186.7	1835.7	119.1	2181.3	170.6	1848.2	0.2077	10.8335	**0.0001**
*uchl1*	177.1	19.9	272.5	21.5	294.8	21.9	267.6	0.1417	1.3368	**0.0033**
*vasa*	479.6	35.4	377.6	28.8	377.0	27.2	390.2	0.0508	−1.0327	0.0819

Low quality is 0–20% eyeing (*N* = 6), Medium quality is 20–80% eyeing (*N* = 27), High quality is 80–100% eyeing (*N* = 15). Values in bold are significant at *P* < 0.05, ND indicates below detection limit. RSQ is square root, RC is regression coefficient, and *P* value is for regression of transcript abundance to eyeing rate for 48 individual samples. RSQ and RC are for normalized data and *P* value is for transformed normalized data

**Table 3:** Group A2 Normalized reads

	Low quality		Medium quality		High quality					
Gene	Mean	SEM	Mean	SEM	Mean	SEM	Mean reads	RSQ	RC	*P* value
Mitochondrial genes										
*mt-atp8*	70,396	3649	105,437	10,120	144,308	19,256	117,095	0.0355	486.1	0.3921
*mt-co1*	187,167	75,666	215,761	24,277	295,887	23,547	241,777	0.0857	1435.7	0.0666
*mt-cytb*	65,679	15,616	109,657	13,106	152,923	14,076	122,408	0.0814	776.0	**0.0426**
*mt-nd4l*	4920	1356	6646	801	10,063	1467	7733	0.0317	36.0	0.2472
*mt-dlp*	9707	4788	17,151	2254	23,632	4410	19,022	0.0540	132.9	0.0839
Nuclear genes										
*agfg1-like*	78.3	2.0	71.8	7.2	85.9	6.2	76.9	0.0084	0.1239	0.7307
*anxa2*	53.8	8.2	73.4	8.0	80.9	9.8	75.1	0.0145	0.1950	0.4492
*apoc1*	1209.5	793.1	2729.3	569.8	1897.9	753.6	2376.8	0.0022	5.5802	0.5534
*atg16l1*	52.8	2.9	70.2	7.4	76.3	7.7	71.5	0.0146	0.1730	0.4387
*bmp10-like*	13.4	1.2	39.3	4.7	35.1	3.8	36.7	0.0130	0.1017	ND
*ctsz*	9929.1	3409.9	13,888.0	1113.6	12,001.9	927.1	13,065.0	0.0010	6.6351	0.4726
*cycB*	14,225.4	3878.4	14,363.5	963.4	15,421.9	1008.5	14,718.0	0.0150	23.2022	0.5360
*dcaf11*	83.8	10.0	147.6	11.4	205.1	14.6	164.3	0.1878	1.1626	**0.0035**
*dglucy*	13.4	1.2	15.5	0.9	14.0	1.1	14.9	0.0007	0.0046	ND
*erich3*	13.4	1.2	15.5	0.9	14.0	1.1	14.9	0.0007	0.0046	ND
*fbxo5*	310.2	123.7	438.7	39.5	691.7	73.1	519.2	0.1702	4.4568	**0.0120**
*galnt3*	354.3	16.0	230.1	20.4	254.5	19.1	244.1	0.0219	−0.5889	0.2481
*gsh-px*	228.0	2.4	484.8	47.2	417.3	54.1	450.1	0.0018	0.4046	0.4661
*gtf3a*	164.5	19.9	247.2	22.1	318.0	39.6	267.6	0.0540	1.2597	0.1150
*haus3*	157.5	49.1	205.8	20.5	283.1	23.4	230.0	0.1106	1.4526	0.0510
*hbb*	6945.0	2128.3	5318.5	1111.5	2745.4	821.5	4515.2	0.0132	−23.9143	0.2282
*ifngr1*	13.4	1.2	15.5	0.9	14.6	1.0	15.1	0.0073	0.0146	ND
*igf-1*	13.4	1.2	23.5	2.7	23.2	2.7	22.9	0.0657	0.1326	ND
*igf-2*	46.9	0.8	72.9	10.1	98.2	25.0	80.4	0.0353	0.5423	0.3252
*igf-3*	13.4	1.2	29.9	3.2	26.2	3.1	27.9	0.0146	0.0750	ND
*igfr1b*	86.0	1.4	93.0	8.1	103.4	7.1	96.2	0.0003	0.0273	0.8610
*il17rd*	506.1	55.0	284.1	27.8	364.1	30.1	321.5	0.0021	−0.2719	0.5848
*impa2*	511.5	186.2	1966.8	199.4	2761.9	315.3	2171.7	0.1689	20.1204	**0.0010**
*ing3*	60.8	4.4	80.0	7.2	101.8	10.5	86.5	0.0261	0.2604	0.2269
*itga7*	18.0	5.8	24.3	2.8	24.7	2.7	24.2	0.0216	0.0772	ND
*kmt5b*	70.8	7.3	61.0	5.9	70.1	6.3	64.6	0.0008	0.0332	0.8023
*krt18*	159.7	0.8	178.3	28.9	116.5	13.6	156.4	0.0022	0.2393	0.8316
*krt8*	95.9	2.6	164.8	39.8	112.0	20.8	143.7	0.0008	0.1936	0.5031
*lin7b*	135.0	28.7	399.2	44.1	493.9	60.0	419.5	0.1175	3.3230	**0.0095**
*mettl3*	13.4	1.2	15.5	0.9	14.0	1.1	14.9	0.0007	0.0046	ND
*mr-1*	316.1	97.1	327.4	20.0	355.0	24.3	336.3	0.0000	−0.0106	0.9443
*mrpl39-like*	54.3	4.5	214.6	26.9	273.2	42.6	227.3	0.0889	1.8588	**0.0126**
*myo1b*	138.0	2.9	68.1	7.7	81.9	7.1	76.0	0.0477	−0.3429	0.1213
*nasp*	157.3	3.3	217.2	17.1	294.0	22.0	240.7	0.0996	1.2183	0.0574
*npm2*	13.4	1.2	16.0	0.8	14.4	1.1	15.4	0.0001	−0.0013	ND
*ntan1*	336.7	52.6	322.3	25.8	356.8	29.8	334.7	0.0051	0.3686	0.8127
*pde4d*	69.2	15.0	87.4	9.6	103.4	8.7	92.0	0.0243	0.2860	0.2916
*pgk1*	173.4	65.0	228.0	12.1	260.1	17.6	236.5	0.0471	0.6028	**0.0465**
*phb2*	54.3	10.9	73.1	5.1	88.9	11.8	77.7	0.0290	0.2436	0.2019
*psmb9*	122.7	63.9	77.6	11.0	51.5	8.0	70.7	0.0015	−0.0826	0.5031
*ptgs2*	630.3	111.9	685.1	68.2	760.7	89.5	708.4	0.0022	0.6635	0.9055
*pyc*	70.7	29.5	72.8	6.2	85.2	7.4	76.9	0.0097	0.1259	0.5615
*ran*	44.4	3.2	41.0	6.2	39.4	4.9	40.6	0.0106	0.1155	ND
*rpl22*	229.6	27.9	566.2	54.1	640.3	134.1	576.2	0.0652	3.9744	**0.0130**
*rpl24*	654.1	0.9	1268.8	156.9	1033.2	285.2	1160.5	0.0065	3.0154	0.6741
*rpl30*	922.6	42.1	1505.6	154.9	1165.1	186.5	1363.0	0.0107	3.2904	0.5562
*rplp1*	40.4	5.7	46.5	5.7	52.3	6.0	48.2	0.0050	0.0781	ND
*rps9*	166.8	11.0	312.3	26.5	264.3	28.1	289.4	0.0135	0.6161	0.1591
*s100a1*	115.8	22.5	154.0	15.7	192.3	15.2	165.3	0.0643	0.7820	0.0981
*samm50*	162.2	42.9	546.5	45.3	650.0	46.0	564.3	0.1262	3.4057	**0.0024**
*sec14l2*	71.6	17.4	88.6	8.0	101.8	10.5	92.3	0.0216	0.2453	0.3916
*senp7*	36.6	6.3	52.3	5.1	72.4	5.9	58.5	0.1001	0.3488	**0.0330**
*ska3*	89.5	20.1	187.6	17.0	253.9	21.2	205.8	0.1664	1.5573	**0.0058**
*slc7a6os*	184.8	14.7	337.6	23.2	393.0	29.6	349.5	0.0732	1.3690	**0.0410**
*smc6*	115.2	2.4	92.9	9.3	103.0	9.4	97.4	0.0014	−0.0669	0.6010
*tfip11*	40.4	5.7	80.5	6.7	113.2	13.8	89.8	0.1357	0.6824	**0.0115**
*tob1*	178.8	18.3	118.6	9.8	122.8	9.9	122.8	0.0399	−0.3867	0.1576
*tubb*	1278.3	495.4	2522.1	174.7	3145.6	253.3	2678.1	0.1492	15.9537	**0.0040**
*uchl1*	177.0	36.0	245.8	18.8	321.2	21.5	268.4	0.1074	1.3353	**0.0348**
*vasa*	645.4	127.0	344.1	27.9	369.0	23.6	366.2	0.0919	−1.7727	0.0507

Low quality is 0–20% eyeing (*N* = 2), Medium quality is 20–80% eyeing (N = 27), High quality is 80–100% eyeing (*N* = 15). Values in bold are significant at *P* < 0.05, ND indicates below detection limit. RSQ is square root, RC is regression coefficient, and *P* value is for regression of transcript abundance to eyeing rate for 44 individual samples. RSQ and RC are for normalized data and *P* value is for transformed normalized data

**Table 4:** Group B Normalized reads

	Low quality		Medium quality		High quality					
Gene	Mean	SEM	Mean	SEM	Mean	SEM	Mean reads	RSQ	RC	*P* value
Mitochondrial genes										
*mt-atp8*	54,603	4919	45,585	3878	68,028	9875	53,384	0.0038	46.3	0.9747
*mt-co1*	153,864	16,034	169,723	16,529	198,231	23,624	167,338	0.0414	494.9	0.0907
*mt-cytb*	72,203	6855	75,366	8870	104,405	12,031	78,783	0.0678	313.2	0.1034
*mt-nd4l*	3332	275	3687	340	5505	618	3830	0.0971	16.5	0.0801
*mt-dlp*	20,327	1675	14,663	1289	13,641	1439	17,041	0.1214	−81.3	**0.0171**
Nuclear genes										
*agfg1-like*	49.7	5.3	39.8	4.1	57.5	6.5	47.2	0.0022	0.0344	0.9480
*anxa2*	44.2	5.0	39.0	4.1	52.4	6.6	43.6	0.0078	0.0610	0.5579
*apoc1*	1478.3	206.3	2151.0	430.9	1207.1	229.3	1691.0	0.0043	3.0190	0.8342
*atg16l1*	47.8	4.5	37.6	4.0	46.4	6.4	43.6	0.0081	−0.0587	0.3115
*bmp10-like*	25.4	2.8	22.8	2.8	32.0	5.6	25.5	0.0193	0.0618	ND
*ctsz*	12,729.3	761.5	9577.1	709.3	7959.5	504.0	10,726.0	0.2075	−53.6418	**0.0003**
*cycB*	11,626.3	654.0	9526.4	636.5	9595.5	637.3	10,482.9	0.0945	−29.7049	**0.0186**
*dcaf11*	131.7	8.9	150.7	9.4	174.1	9.4	146.0	0.0942	0.4223	**0.0158**
*dglucy*	17.0	3.4	13.1	1.8	12.8	1.4	14.8	0.0159	−0.0500	ND
*erich3*	11.5	1.0	12.1	0.9	12.8	1.4	12.0	0.0123	0.0160	ND
*fbxo5*	647.2	61.3	660.8	51.1	683.6	65.6	658.5	0.0101	0.8246	0.3339
*galnt3*	192.2	13.7	170.9	16.3	196.9	14.3	184.8	0.0013	−0.0760	0.6643
*gsh-px*	441.4	41.5	354.8	30.5	353.8	57.5	393.6	0.0262	−0.9181	0.2192
*gtf3a*	205.5	14.6	200.0	15.4	235.9	12.7	208.4	0.0227	0.3200	0.1589
*haus3*	157.0	13.1	153.5	14.2	185.6	14.9	160.4	0.0188	0.2699	0.2781
*hbb*	3189.6	931.9	6115.2	1972.1	1474.8	705.7	4025.3	0.0002	−3.2748	0.7471
*ifngr1*	11.7	1.0	12.0	1.0	12.8	1.4	12.0	0.0038	0.0091	ND
*igf-1*	18.2	1.9	17.4	1.8	19.9	2.7	18.2	0.0010	0.0086	ND
*igf-2*	37.7	4.2	25.0	3.1	35.4	9.3	32.5	0.0126	−0.0727	0.2445
*igf-3*	17.8	1.9	17.0	1.6	17.9	2.6	17.5	0.0013	0.0096	ND
*igfr1b*	60.9	5.4	56.5	4.7	75.1	8.4	61.6	0.0173	0.1043	0.5230
*il17rd*	212.9	15.8	193.2	15.0	254.3	28.3	212.2	0.0147	0.2961	0.5738
*impa2*	1384.4	100.3	1668.8	118.1	1894.1	123.3	1578.4	0.1558	6.5165	**0.0009**
*ing3*	67.0	4.6	71.1	5.7	81.3	7.4	71.0	0.0324	0.1365	0.2700
*itga7*	15.3	1.2	17.2	1.8	20.9	2.8	17.0	0.0503	0.0525	ND
*kmt5b*	45.8	4.1	43.1	3.4	60.8	8.3	47.2	0.0315	0.1125	0.2804
*krt18*	173.7	23.1	181.3	21.5	86.4	14.6	162.0	0.0400	−0.6560	0.2239
*krt8*	137.8	16.7	150.6	15.8	87.1	11.6	134.2	0.0337	−0.4345	0.2211
*lin7b*	314.3	27.9	294.8	23.6	276.1	25.7	300.5	0.0039	−0.2336	0.8913
*mettl3*	11.5	1.0	11.7	1.0	12.8	1.4	11.8	0.0046	0.0102	ND
*mr-1*	242.6	11.9	196.9	13.0	201.3	17.3	218.2	0.0699	−0.5097	**0.0435**
*mrpl39-like*	210.2	24.4	273.6	27.8	263.3	16.6	243.4	0.0759	1.0183	**0.0092**
*myo1b*	48.5	4.3	43.9	3.1	54.5	7.1	47.7	0.0028	0.0317	0.9864
*nasp*	151.8	10.7	176.1	11.1	205.8	14.0	170.1	0.1473	0.6445	**0.0030**
*npm2*	12.2	1.0	11.7	1.0	12.8	1.4	12.1	0.0001	0.0015	ND
*ntan1*	231.6	14.9	186.0	15.7	206.3	13.3	209.9	0.0305	−0.3887	0.1795
*pde4d*	52.3	4.6	49.6	6.0	62.5	8.1	53.0	0.0091	0.0750	0.7286
*pgk1*	176.9	9.2	179.8	7.3	206.0	14.0	182.9	0.0353	0.2448	0.2972
*phb2*	45.0	2.9	49.9	3.7	60.7	8.1	49.5	0.0927	0.1713	0.0600
*psmb9*	52.9	7.5	54.8	6.6	32.1	3.7	50.1	0.0182	−0.1367	0.7264
*ptgs2*	413.4	39.1	328.0	42.5	523.1	88.8	398.9	0.0135	0.7859	0.5895
*pyc*	44.0	4.4	43.8	4.1	54.5	5.7	45.7	0.0185	0.0862	0.3774
*ran*	29.2	3.4	27.5	3.4	23.8	2.8	27.6	0.0033	−0.0276	ND
*rpl22*	664.3	66.2	590.6	52.8	471.7	38.7	604.0	0.0379	−1.6919	0.2635
*rpl24*	1029.6	132.7	900.4	93.3	478.0	55.6	888.1	0.0750	−4.7192	0.0655
*rpl30*	1473.7	147.7	1374.3	124.6	779.6	82.3	1319.9	0.0819	−5.8789	**0.0342**
*rplp1*	33.6	2.7	30.9	2.4	35.9	3.3	32.9	0.0059	0.0287	ND
*rps9*	264.9	18.9	278.0	20.2	179.8	10.6	255.7	0.0449	−0.6094	0.1525
*s100a1*	110.2	10.0	99.2	9.8	120.2	14.3	107.7	0.0003	0.0243	0.9801
*samm50*	333.4	21.0	322.3	21.8	409.3	18.5	341.8	0.0365	0.5987	0.2896
*sec14l2*	52.5	4.3	43.0	5.0	61.7	6.8	50.4	0.0052	0.0513	0.9647
*senp7*	43.2	3.8	54.5	4.1	59.3	4.8	50.2	0.1060	0.1956	**0.0036**
*ska3*	193.4	14.7	176.4	12.0	206.9	11.1	189.1	0.0033	0.1109	0.4935
*slc7a6os*	272.4	15.0	293.4	18.5	283.3	9.9	282.3	0.0087	0.2153	0.3751
*smc6*	80.3	5.2	63.8	4.5	77.0	7.2	73.4	0.0282	−0.1270	0.1358
*tfip11*	55.0	5.0	63.8	4.6	65.4	5.3	60.1	0.0575	0.1697	**0.0055**
*tob1*	96.7	9.0	79.7	5.8	75.5	8.7	86.7	0.0454	−0.2446	0.1939
*tubb*	2223.9	101.4	2133.1	122.1	2264.9	130.4	2195.9	0.0001	−0.1152	0.9580
*uchl1*	199.8	10.4	220.1	15.8	292.2	13.1	223.0	0.1781	0.8769	**0.0063**
*vasa*	241.6	15.5	216.4	13.1	291.0	43.1	240.2	0.0068	0.2202	0.9890

Low quality is 0–20% eyeing (*N* = 27), Medium quality is 20–80% eyeing (*N* = 23), High quality is 80–100% eyeing (*N* = 10). Values in bold are significant at *P* < 0.05, ND indicates below detection limit. RSQ is square root, RC is regression coefficient, and *P* value is for regression of transcript abundance to eyeing rate for 60 individual samples. RSQ and RC are for normalized data and *P* value is for transformed normalized data

**Table 5:** Summary of gene transcripts with significant correlations between transcript abundance and eyeing rate

		Group	
Gene	A1	A2	B
*mt-cyb*	P	P	–
*mt-dlp*	–	–	N
*dcaf11*	P	P	P
*impa2*	P	P	P
*mrpl39-like*	P	P	P
*senp7*	P	P	P
*tfip11*	P	P	P
*uchl1*	P	P	P
*lin7b*	P	P	–
*pgk1*	P	P	–
*rpl22*	P	P	–
*samm50*	P	P	–
*ska3*	P	P	–
*slc7a6os*	P	P	–
*tubb*	P	P	–
*apoc1*	P	–	–
*galnt3*	N	–	–
*gsh-px*	P	–	–
*gtf3a*	P	–	–
*ing3*	P	–	–
*krt18*	P	–	–
*myo1b*	N	–	–
*psmb9*	P	–	–
*rpl24*	P	–	–
*rsp9*	P	–	–
*tob1*	N	–	–
*nasp*	P	–	P
*fbxo5*	–	P	–
*ctsz*	–	–	N
*cycB*	–	–	N
*mr-1*	–	–	N
*rpl30*	–	–	N

Significant correlations between transcript abundance and eyeing rate as identified by regression analysis. P indicates a significant positive correlation and N indicates a significant negative correlation, *P* < 0.05

In Group A1 regression analysis identified 25 nuclear and one mitochondrial gene with transcript levels correlated with eyeing rate (Table 2; Fig. 2a). Twenty-two of the nuclear genes and the mitochondrial gene, *mt-cyb*, had increased transcript abundance with increased survival and three decreased. The coefficient of determination ( R[2]) values for normalized untransformed data were at or below 0.2269 for all genes. Three genes, *impa2*, *linb7*, and *mrpl39-like* had over three times more transcripts in the high-quality eggs (80–100% eyeing) than in the low-quality eggs (0–20% eyeing). There were five genes in which the medium-quality eggs (20–80% eyeing) had the highest and one the lowest number of reads, and *apoc1* had about three times more abundant reads than either the low- and high-quality eggs which had read amounts similar to each other. The numerical means for all the mitochondrial genes in the high-quality eggs were 46–105% above the low-quality eggs.

In Group A2 there were 14 nuclear genes and one mitochondrial gene with correlated transcript abundance and eyeing rates (Table 3; Fig. 2b). All but *fbxo5* were also significant for A1 (Table 5). Transcript abundance and eyeing rates were positively correlated for all DEGs and R^2^ values were at or below 0.1878. As in A1, *impa2*, *linb7*, and *mrpl39-like* had over three times more transcripts in the high-quality eggs than in the low-quality eggs, as did *samm50* in A2. There were no DEGs in which the medium-quality eggs had the highest or lowest number of reads. The numerical means for all the mitochondrial genes in the high-quality eggs were 58–143% above the low-quality eggs.

In Group B Regression analysis identified 11 nuclear and one mitochondrial gene with transcript levels correlated with eyeing rate (Table 4; Fig. 2c). Transcript abundance of seven of the nuclear genes increased with eyeing rate whereas the remaining four along with the mitochondrial gene *mt-dlp*, decreased. Six of the nuclear genes were also significant for both A1 and A2, *nasp* was also significant for A2, whereas the remaining 4 and the mitochondrial gene *mt-dlp* were only significant for B (Table 5). Furthermore, transcript abundance of all the DEGs shared with A1 or A2 were positively correlated with eyeing rate whereas the remaining transcripts including *mt-dlp*, were all negatively correlated. The R^2^ values were at or below 0.2075 for the DEGs and differences among low-, medium- and high-egg quality family means were less than 2-fold for all DEGs. The numerical means for all the mitochondrial genes other than *mt-dlp* were 25–65% greater in the high-quality eggs than the low-quality eggs.

## Discussion

### Assay performance

The nCounter analysis data system was selected with possible use by hatchery managers in mind. Nanostring Technologies designs the custom CodeSets for nCounter® analysis based on submitted target sequences and conducts the genomic analyses required to avoid non-specific hybridization, and provides free software, nSolver, to quality check, normalize, and analyze the data. In addition, the system can include over 800 genes and new probes can easily be exchanged in the CodeSet. In our assay the mean raw reads for a given gene were consistent across the three groups or populations with an average CV of 18.6% (Additional file 1: Table S4D) comparing among the three groups. The high abundance of reads for the five mitochondrial genes limited read values for many nuclear genes with 14 below acceptable limits including two of the reference genes *ef1a* and *ppia* (Additional file 1: Table S4D). The CodeSet can be designed to attenuate high abundance genes to alleviate this problem. The cause for apparent instability of *b-actin* in A1 is not known but it was also found to be unstable among the 20 samples from A1 previously analyzed by RNA-Seq [27]. Nevertheless, the reliability of reference genes can be questionable with egg quality in which the proportion of mitochondrial and nuclear transcripts can vary with the quality of the eggs [27] and the efficiency of methods to capture polyadenylated transcripts can vary for shorter poly(A) tail lengths as with stored maternal mRNAs. Whereas the majority of cytosolic nuclear transcripts in most cells are polyadenylated with a poly(A) tail greater than 80 nucleotides [37, 38], stored maternal nuclear transcripts possess a short poly(A) tail of around 15–40 nucleotides that are elongated to over 80 nucleotides through cytoplasmic polyadenylation during activation [37, 39–42]. Oligo(dT) capture approaches in general are not very efficient at capturing mRNAs with shorter poly(A) tails [39, 43, 44]. Assay performance and normalization may be improved with the use of an RNA spike-in as described by Bettegowda et al. [45] for use in bovine oocytes. An RNA with a poly(A) tail with a minimum of 25 nucleotides that would likely be efficiently capture by most oligo(dt) methods, and can be used starting at homogenization, should be considered.

### Eyeing rate and early embryonic viability

Previous studies have characterized early mortality in line A. We characterized mortality in the 20 families from Group A1 when used in our previous analysis of transcript abundance [27]. In these 20 families almost all the mortality observed by eyeing, took place between the 8- and 32-cell stages. Stoddard et al. [36] studying the same line determined most of the mortality in subfertile embryos took place by the second cleavage interval. Although the timing was slightly different, in both studies most of the mortality took place before the MBT and therefore before ZGA. Although the timing of the ZGA has not been determined for rainbow trout, the major wave of ZGA in most fish investigated takes place after the 64-cell stage [46–50]. Furthermore, the fish species investigated develop more rapidly than rainbow trout and in general the number of cleavage divisions completed before ZGA is greater in animals that develop more slowly [51]. We normally see a more prolonged period of mortality in embryos from our NCCCWA broodstock as observed with the families selected for the present study. Unfortunately, we sampled the embryos in Group B earlier than when we sampled A1, and therefore were not able to determine what percentage of mortality took place before the 32-cell stage which was the end of most pre-eyeing mortality in A1. Nevertheless, there was considerable mortality between fertilization and the 8-cell stage; the 8-cell stage and the 16–32-cell stages at about 20 h; ~ 20 h and streak; and between streak and eyeing (Table 1). As mentioned, the streak rate is a very rough estimate, but the values support at least about 28% of mortality by eyeing took place after the ZGA, suggesting the causes for mortality are likely, at least in part, different for the A and B populations.

### Gene expression

#### Mitochondrial genes

The rainbow trout mitochondrial genome encodes 13 polypeptides, two rRNAs, 22 tRNAs and a non-coding region [52]. All 13 polypeptide genes and the mt-dlp region transcripts were found to be reduced to a similar degree in eggs with low eyeing rates by Ma et al. [27]. The present assay included five mitochondrial genes, one for a polypeptide from each of the four complexes of the electron transport chain, and the non-coding mt-dlp region. In the zebrafish, mitochondria are required for oxidative phosphorylation to generate ATP for early cleavage events as the maternal ATP pool is insufficient to sustain the proteasomal pathway required for protein degradation needed to advance beyond the 32-cell stage [53]. Only two of the mitochondrial genes were found to be differentially expressed among the three studies, with *mt-cyb* being increased with eyeing rate in A1 and A2 and *mt-dlp* transcripts being negatively correlated with eyeing rate in B (Tablse 2, 3, 4, 5). The *P*-values reported in Ma et al. [27] for the differences in expression for the mitochondrial genes were generally much higher than for the nuclear genes selected to be in the present assay, which may also contribute to why a greater proportion of the selected nuclear genes were identified as DEGs in the present groups. Nevertheless, all the mitochondrial genes in all groups trended towards increasing with eyeing rate except for *mt-dlp* transcripts in group B, consistent with decreased mitochondrial gene expression being a common feature of eggs with reduced developmental competence. Monitoring a suite of mitochondrial genes for small but similar differences in expression may be a reliable approach to identifying eggs with compromised mitochondrial function.

#### Nuclear genes

A total of 48 nuclear genes were at detectable levels for all three groups. Transcript abundance was correlated with eyeing rate in 30 of these nuclear genes in at least one of the three groups (Table 5; Fig. 2a-c). All the DEGs except *ctsz*, *cycB* and *mr-1* were among the 49 nuclear genes included in the assay because they were identified as DEGs in our previous RNA-seq study on Group A1. Of the 17 genes included in the assay that had previously been reported as DEGs in studies of rainbow trout other than just Ma et al. [27] [28–30], eight were identified as a DEG in at least one of the present groups, seven including *igf-2* were not significantly altered, and two (*igf-1* and *npm2*) were below the detection limit in all groups. Therefore, among the genes that were represented at detectable levels, about half of the transcripts selected from our previous study on Group A1, and half of the transcripts identified from studies by different investigators, served as markers for egg quality in at least one of our study groups supporting a fair degree of overlap in genes altered with egg quality among rainbow trout populations.

The highest number of nuclear DEGs, 25, was in Group A1 (Table 2; Fig. 2a) as would be expected since most of the genes in the assay were identified as DEGs using samples from this group [27]. About half as many genes were DEGs in A2 (Table 3; Fig. 2b), with 13 of the 14 being DEGs in both A1 and A2 and only *fbxo5* significant in A2 and not A1 (Table 5; Fig. 2ab). All 26 genes identified as DEGs for either A1 or A2 in the present study, including *fbxo5*, were DEGs in Ma et al. [27]. Transcript levels of all 14 genes that were DEGs in both A1 and A2 were positively correlated with eyeing rate and only *myo1b*, *galnt3* and *tob1* were negatively correlated in A1. The mitochondrial gene *mt-cyb* was also increased with eyeing rate in both A1 and A2. This uniformity in the profiles of maternal transcript dysregulation with poor egg quality for both year classes for population A supports a consistent cause for reduced egg quality in this line. A consistent pattern of dysregulation increases the chances that further investigation can elucidate physiological reasons and an underlying condition for variation in egg quality among females of a broodstock. Again, these transcripts derive from a global transcriptome analysis of egg quality in this line [27].

Despite being from a different line than the one from which most of the genes in the assay were selected, Group B (Table 4; Fig. 2c) had a similar number of nuclear genes with expression levels that correlated with eyeing rate as A2, 11; of which seven were shared with A1 and A2 (Table 5; Fig. 2a-c). The four genes that were unique DEGs to Group B were also the only four that decreased in abundance with increased eyeing rate. One of the four genes that were unique DEGs to Group B, *rpl30*, was identified as a DEG in A1 following RNA-Seq analysis [27]. Therefore, whereas all the genes identified as DEGs in A1 and A2 were previously reported in [27], Group B had three genes that were not. These genes included the two highest expressed nuclear genes in the assay*, cycB* and *ctsz*. Transcript abundance of *cycB* increased with post-ovulatory ageing and malformation rate at yolk sac resorption, but not survival at eyeing [29]; and *ctsz* increased with post-ovulatory ageing and decreased with eyeing rate [30]. The abundance of the remaining unique DEG, *mr-1*, decreased in eggs from fish induced to ovulate with hormone injections; a treatment that also results in more deformed embryos at yolk sac resorption [28]. Whereas *ctsz* and *cycB* generally increased with measures or treatments associated with reduced egg quality among the studies, *mr-1* which increased with eyeing rate in our studies, decreased in response to hormone induced spawning.

Two genes, *tubb* which increased with eyeing rate in A1 and A2, and *krt18*, which increased with eyeing rate in A1, were also identified as DEGs with eyeing rate by Aegerter et al. [30]. Bonnet et al. [28] found *rpl24*, *myo1b*, and *apoc1*, which were DEGs in A1, to have altered expression in response to treatments that changed spawning time, which were in turn shown to increase malformation rates at yolk sac resorption. However, the direction of the effects did not agree among the studies. Transcripts for *tubb* were increased with eyeing rate in both studies. On the other hand, *krt18* decreased with increasing eyeing rate in Aegerter et al. [30] but was increased with increased egg eyeing rate in Group A1. Nevertheless, the R^2^ was very low for A1, 0.0453 (Table 2), and the families with medium fertility had the highest mean transcript levels in Groups A1, A2, and B (Tables 2, 3, 4; Fig. 2a-c). Transcript abundance for *myo1b* was negatively correlated with eyeing rates in the present study and increased in eggs from fish induced to spawn with photoperiod shifting [28], whereas *apoc1* and *rpl24* were positively correlated with eyeing rates in the present study but were increased in eggs of fish injected to spawn with a gonadotropin-releasing hormone analog [28].

Many genes that were not significantly correlated with eyeing rate or were below detection in the present study were identified as DEGs in the previous studies of egg quality in rainbow trout. Genes for which no correlation between transcript abundance and eyeing rates were found in the present study included genes of the IGF system, *igf-2* and *igfr1b.* Aegerter et al. [29] found *igf-1,* which was below detection in the present assay, and *igf-2,* to be associated with eyeing rate, and although *igfr1b* was not associated with eyeing rate, it was decreased with increased malformation rates at yolk sac resorption. Transcript abundance of *krt8* and *ptgs2* were not altered in our groups although they were negatively correlated with eyeing rate by Aegerter et al. [30]. Transcript abundance of *npm2* and *igf-1* were increased with eyeing rate in the same study but were below detection in our assay. Detection limits for several of these genes in the present study impaired a comparison among studies. Transcript abundance of *pyc* and *ntan1* were not altered in our groups but were increased in fish exposed to a shifted photoperiod and hormone injection to affect time of spawning, respectively [28].

In all, differences in transcript profiles can be found among all studies and populations in which a relation between egg quality and maternal transcriptome have been investigated in rainbow trout. The bases for these differences among studies are not known but there were differences in when mortality took place and treatments associated with decreased developmental competence among the studies that likely influenced or were influenced by the transcript profiles. In the present study almost all the mortality that would take place by eyeing likely took place by 24 h in line A based on studies by Ma et al. [27] on Group A1 and Stoddard et al. (2015), looking at an earlier year class of the same line. Later mortalities in Group B suggests at least some different factors associated with mortality before eyeing. Further analyses including time of mortality within population B families may suggest if there are transcript profiles associated with early and later embryonic mortality. Aegerter et al. [29, 30] used post ovulatory ageing to decrease egg quality whereas it was purposely avoided as in the present study. Genes identified as DEGs in the studies on post ovulatory ageing that were not DEGs in the present study or had opposite correlations might suggest those genes that are more specific or responsive to post ovulatory ageing. This includes *krt8*, *krt18*, *ptgs2*, and possibly *igf-2* or the IGF system in general. Different expression profiles between the present study and the study by [28] may not be surprising considering the transcript levels in [28] were in response to treatments that were in turn associated with increased malformations at yolk sac resorption as a measure of egg quality. Thus, the transcript profiles in this previous study were not only not in association with the same egg quality metric as in the present study, they were not even in direct association to that egg quality metric, malformations at yolk sac resorption. Nevertheless, the differences in responses of many genes among the studies shows these transcripts cannot be used as general markers of egg quality but does not negate important roles for these transcripts in embryo development.

The identification of six DEGs shared among our three groups, *dcaf11*, *impa2*, *mrpl39_like*, *senp7*, *tfip11* and *uchl1*, supports some consistency among populations in transcripts that are altered with the egg quality metric eyeing rate. Considering the six genes were identified as DEGs in our previous study that included in-depth genomic analyses within the context of all the identified DEGs in Group A1 [27], we will not again discuss possible functions of these genes in detail. The genes do however belong to both disparate and overlapping functional pathways. Several are involved in proteolysis with *dcaf11* and *uchl1* functioning in ubiquitin pathways, and *senp7* and *impa2* having hydrolase activity. On the other hand, *tfip11* is involved in RNA processing and *mrpl39* at least, is involved in mitochondrial function. Unfortunately, they were not included as target genes in previous studies on rainbow trout in which eyeing rate was a metric [29, 30]. Nevertheless, they were dysregulated in all three of our groups and therefore are the most promising as markers for poor egg quality. Unfortunately, even for these genes the regression coefficients and R^2^ were low and therefore the expression of any one gene would not be effective at predicting quality of a single batch of eggs. As previously suggested, a suite of genes would likely be required to identify eggs of different developmental competence [20, 23]. Still more data would be required if the bases or causes of gene dysregulation, or more specific quality outcomes are to be predicted.

## Conclusions

The present study confirmed DEGs for eyeing rate identified through a comparison of a small number of individuals by RNA-Seq can be extended to the broader population. However, for each of the DEGs identified there is considerable variability in transcript abundance among eggs of similar quality and low correlations between transcript abundance and eyeing rate, making it highly improbable to predict the quality of a single batch of eggs based on transcript abundance of just a few genes. The DEGs were more consistent among these two year classes of the same line than between either of these two groups and Group B which is from a different line. Greater similarity in dysregulated transcripts across year classes within the same line than among lines suggests patterns of transcriptome dysregulation may provide insight into causes of decreased viability specific to a hatchery population.

Although not as similar as among year classes within the same line, there appears to be commonality in genes that are dysregulated even among diverse populations and metrics for evaluating egg quality. Transcript abundance of over half of the 17 genes in the assay that were identified as DEGs among rainbow trout eggs of disparate quality based on a range of egg quality metrics in populations other than those in the present study, were found to be correlated with eyeing rate in at least one of our study groups, although not always in the same direction as in the previous studies. There were six genes with transcript abundance correlated with eyeing rate in all three groups, *dcaf11*, *impa2*, *mrpl39_like*, *senp7*, *tfip11* and *uchl1,* and therefore have the greatest potential to serve as general markers for egg quality among those in our current study.

Other than the mitochondrial genes, the genes selected for the present study were primarily based on magnitude of response and statistical differences reported in previous studies. Despite this lack of focus in the genes selected, many of these genes were found to be differentially expressed in response to differences in egg quality in our other populations; and similarities and differences in expression profiles among the studies and our groups were identified and useful inferences were deduced. Future assays need to be designed for investigating specific pathways involved with egg quality parameters and designed to address dysregulation in specific hatchery populations.

## Methods

### Sample collection

Eggs were collected from 192 individual two-year-old broodstock rainbow trout from the May even-year selective breeding program at Troutlodge Inc. Sumner, WA, USA (Group A1); and 142 individuals from the May odd-year line (Group A2); and 325 individuals from the 2015 NCCCWA line (Group B). Samples were collected for Groups A1 and A2 one year apart, following the same procedures as described in Ma et al. [27] for Group A1. Briefly, approximately 90 unfertilized eggs from each female were frozen in liquid nitrogen for mRNA analysis and 50 unfertilized eggs were fixed in modified Davidson’s fixative [54] to be examined for overripening or other abnormalities. Sperm harvested from a single neomale was used to fertilize the remaining eggs from each female and the collected semen from each male was used for two or three crosses. Eyeing rate was evaluated at about 250 ATUs (sum of mean daily water temperature in degrees Celsius) (Fig. 1ab). About 25–60 embryos per family in Group A1 were fixed in Stockard’s solution [55] at about the 32-cell stage. Enumeration of embryos reaching each cleavage stage was used to assess viability. The data for Group A1 are reported in Ma et al. [27], and samples to evaluate early embryonic survival were not collected from Group A2. Sample collection for Group B followed similar procedures as for Groups A1 and A2 with minor exceptions. Batches of unfertilized eggs or spawns with evidence of overripe eggs or other abnormalities were not collected, but a 5-ml sample of unfertilized eggs from retained lots were also collected in neutral-buffered formalin to be examined again for elimination of batches of eggs with such traits. Two 5-ml samples of about 50 unfertilized eggs each were collected from each female and immediately frozen in liquid nitrogen for mRNA analysis. Semen derived from a single neomale sire was used to fertilize eggs from a single female, so no half-sibling crosses were generated to evaluate sperm quality. Eyeing rate was determined at about 250 ATUs (Fig. 1c). About 40 embryos from each family were collected after 19–20 h of incubation at 10 °C to be evaluated for early embryonic viability as described with Group A1 (Table 1; Additional file 1: Table S2). In addition, at approximately 10 days post fertilization 10 embryos with normal coloration from each spawn were fixed in Davidson’s fixative to provide a rough estimate of survival later in development (Table 1; Additional file 1: Table S2). Only unfertilized eggs and early cleavage stage embryos were sampled for this study and therefore no animals were euthanized.

### Selection of rainbow trout females for mRNA analysis of unfertilized eggs

Egg samples were selected for mRNA analysis based on eyeing rate (Fig. 1d-f) with 130–218 individuals from each family evaluated at eyeing for Group A1, 54–207 for Group A2, and all eggs for Group B (Fig. 1a-c). Unfertilized eggs, eggs with precipitated yolk in response to egg shocking, and those with poorly developed eyes were classified as not eyed for Groups A1 and A2, whereas poorly developed eyes were not a criteria in Group B. Low-quality eggs were those with less than 20% viability at eyeing, medium-quality eggs were those with 20–80% viability, and high-quality eggs were those with above 80% viability. Eggs from the 20 females from Group A1 used in our previous RNA-seq study [27] were included in the present study. Only six females in A1 and two females in A2 yielded eying rates below 30% so eggs from all these females were included in the study. The samples from the medium and high viability families for all groups, and the low viability families for group B, were selected to provide a range of eying rates.

### Assessment of early embryo development

Embryo development for females from Group B selected for mRNA analysis were examined for early embryonic development at 19–20 h post fertilization and about 10 days post fertilization. The embryos fixed in Stockard’s solution at about 19–20 h post fertilization were immersed in 0.5% methylene blue overnight before evaluation. The cell number of each of 25 embryos per family was counted or confirmed to be greater than 32 cells using a stereo microscope (Nikon SMZ660)*.*

The ten embryos from each family with normal coloration collected at ten days post fertilization and fixed in Davidson’s fixative were examined by eye to enumerate the embryos that had reached development of the neural keel [56]. The percentage of embryos reaching this stage, was then adjusted by the estimated percent of eggs that did not have normal coloration or were white in appearance indicating yolk precipitation, based on a rough visual estimate of the eggs in the batch, and this was recorded as the streak rate (Table 1; Additional file 1: Table S2). The streak rate was meant as a hatchery tool and is a very rough estimate of early embryonic survival.

### RNA isolation

Total RNA was isolated from a pool of 50 eggs per fish as described in Ma et al. [27]. Briefly, Eggs were homogenized while frozen in Tri Reagent (Sigma, St. Louis, MO) using a Qiagen Retsch MM300 TissueLyser (Retsch Inc., Haan, Germany). Total RNA was isolated using the manufacturer’s suggested protocol. The aqueous phase was separated from the organic phase using Phase Lock Gel tubes (5 PRIME, Inc., Gaithersburg, MD) and Phase Separation Reagent (Molecular Research Center, Cincinnati,OH). Isolated RNAs were purified further by a lithium chloride precipitation and then treated with DNase. Polyadenylated RNA was then collected using Oligotex mRNA Mini-Kits (Qiagen, Germantown, MD).

### Transcript abundance analysis

The study used an nCounter analysis data systems assay comprised of 65 transcripts previously identified as being associated with measures of egg quality in rainbow trout, along with four reference genes (Additional file 1: Table S1). Annotated sequence data for each gene was submitted to Nanostrings Technologies (Seattle, WA) for CodeSet design. Nanostring Technologies designed custom CodeSets for nCounter® analysis using the submitted target sequences and rainbow trout transcriptome sequence data to avoid non-specific hybridization. CodeSet details are provided in Additional file 1: Table S1. Transcript measurement was conducted following the nCounter analysis system workflow protocols. Ten ng of mRNA was used for each sample for A1, 7.6 ng for A2, and 6.15 ng for B. The raw data before normalization are provided in Additional file 1: Tables S4A-D and normalized data used for transcript abundance analysis are presented in Additional file 1: Tables S5A-C.

### Normalization of nCounter transcript abundance data

The nSolver Analysis Software (V2.0) was used for normalization of transcript abundance data. The average geometric mean of positive spike-in RNA controls was used across all samples to normalize for technical aspects of assay performance. Although four reference genes were included in the assay, both *ef1a* and *ppia* were below detection limits and *b-actin* was not stable in Group A1 samples. Therefore, the geometric mean of *g6pd* and *b-actin* were used for normalization in Groups A2 and B, and *g6pd* alone for Group A1. The assay included both mitochondrial and nuclear genes. The mean raw reads per gene was 6107, however, the mean raw reads for the five mitochondrial genes was 76,186 whereas the mean raw reads for the nuclear genes was 632 (Additional file 1: Table S4D). The high reads for the mitochondrial genes overwhelmed the nuclear genes resulting in 12 genes in addition to two of the reference genes being less than the geometric mean plus two standard deviations of the negative controls in at least one of the groups (Additional file 1: Table S4D). The average CV among the three studies for the raw reads for the individual genes was 18.6% (Additional file 1: Table S4D).

### Statistical analysis

Statistical analyses were conducted separately for the three studies and for nuclear and mitochondrial transcripts. The transcripts selected for the assay were based primarily on studies in which low egg quality was classified as having eying rates below 20% and high egg quality as having eyeing rates above 80% [27, 29, 30]. Families were therefore classified as having low-quality eggs, 0–20% eyeing, medium-quality eggs, 20–80% eyeing, and high-quality eggs, 80–100% eyeing in the present study and mean and standard errors of the mean (SEM) are presented (Tables 2, 3, 4; Fig. 2 a-c). Unfortunately, Group A1 had only six and Group A2 had only two samples with eyeing rates below 30%, hampering the ability to use multivariate analysis, and therefore regression and analysis of variance were used to test for correlations and measure the effect of gene transcript abundance on the egg quality phenotype eyeing rate.

Prior to conducting statistical data analysis, the datasets were transformed as needed to meet normality of distribution and equal variance requirements of the statistical data analyses. We performed log10, arcsin and square-root data transformation, and assessed the fit of the transformed data to normal distribution using statistical tests available in the Procedure Univariate from SAS software [57]. Then, to quantify the correlation or impact of gene transcript abundance on the egg quality phenotype eyeing rate, we performed two types of statistical data analysis: First, we estimated the regression coefficient of the continuous phenotype eyeing rate on gene transcript abundance using the Procedure Regression from the SAS software [57]. Second, we performed analysis of variance to determine whether each discretized eyeing rate phenotype (i.e., low, medium, high) was associated with a distinct level of gene transcript abundance using the Procedure GLM from the SAS software [57]. For both analyses, the threshold significance level was set at the nominal *P*-value of *P* < 0.05. Although data for all genes were analyzed, those with average raw read levels less than that of the geometric mean plus two standard deviations of the negative controls for that group, were considered unreliable and therefore below detection. The geometric means plus two standard deviations for the negative control raw reads were 40.3, 38.3 and 30.6 for Groups A1, A2, and B respectively (Additional file 1: Tables S4A-C).

## Supplementary Information

**Additional file 1: Table S1.** Gene abbreviations and sequence of gene coding regions targeted for Nanostring nCounter CodeSet. **Table S2.** Assessment of early embryo development in each Group B selected family. **Table S3A.** Regression analysis of mitochondrial gene transcript abundance and eyeing rate. **Table S3B.** Regression analysis of nuclear gene transcript abundance and eyeing rate. **Table S4A.** Group A1 individual raw reads from Nanostrings assay. **Table S4B.** Group A2 individual raw reads from Nanostrings assay. **Table S4C.** Group B individual raw reads from Nanostrings assay. **Table S4D.** Summary data for individual raw reads from Nanostrings assay. **Table S5A.** Group A1 individual normalized reads. **Table S5B.** Group A2 individual normalized reads. **Table S5C** Group B individual normalized reads.

## Data Availability

All data generated or analyzed during this study are included in this published article and its supplementary information files. The probes used in the current study were designed based on a previously published dataset ([27]; SRA:SPR108797).

## Abbreviations

ZGA: Zygotic genome activation
MBT: Mid-blastula transition
DEG: Differentially expressed gene
Poly(A): Polyadenylic acid
ATUs: Accumulated thermal units
NCCCWA: National center for cool and cold water aquaculture. Gene name abbreviations are presented in Additional file 1: Table S1.
